# Evaluating the Effects of Anode Porous Transport Layer on the Performance and Durability of Anion Exchange Membrane Electrolyzers

**DOI:** 10.1021/acsomega.6c01506

**Published:** 2026-05-17

**Authors:** Saad Intikhab, Alexandra Oliveira, Kimberely S. Reeves, Haoran Yu, Yushan Yan, Shaun M. Alia

**Affiliations:** 1 Chemical and Material Sciences Center, 53405 National Laboratory of the Rockies, 15013 Denver West Parkway, Golden, Colorado 80401, United States of America; 2 Department of Chemical and Biomolecular Engineering, 5972 University of Delaware, 221 Academy Street, Newark, Delaware 19716, United States of America; 3 Center for Nanophase Materials Sciences, 6146 Oak Ridge National Laboratory, 1 Bethel Valley Road, Oak Ridge, Tennessee 37830, United States of America

## Abstract

As anion exchange membrane systems have emerged as a competitive low temperature electrolysis technology, research has expanded to other components and device integration. In this study, nickel (Ni) and stainless steel (SS)-based porous transport layers (PTLs) are investigated in membrane electrode assemblies (MEAs). Compared to MEAs using Ni, the SS PTL shows higher performance due to less kinetics and residual loss and possibly due to a combination of iron mobility improving oxygen evolution reactivity and electron conduction pathways, as well as higher porosity increasing site access. Voltage decay rates of approximately 144 and 115 μV/h, respectively, for the Ni and SS PTLs are found, although the long-term durability and lifetime implications are convoluted. Voltage breakdown analysis confirms that both PTLs saw significant increases in residual loss possibly due to catalyst/PTL property changes that affected electronic, ionic, and mass transport pathways. For the Ni PTL, a higher proportion of the losses were due to cell kinetics; comparatively, more of the SS PTL losses were due to increases in the high frequency resistance. The experimental findings presented here provide insights on the impact of the PTL materials and their properties.

## Graphical abstract

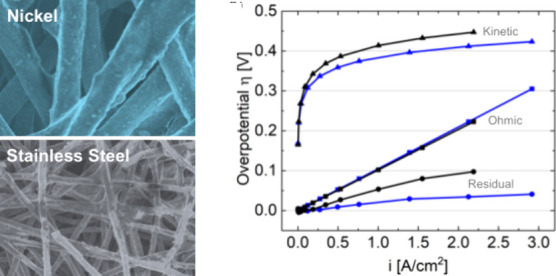

## Introduction

While hydrogen is a relatively small contributor to energy pathways and is primarily produced through steam methane reforming, hydrogen is a critical feedstock in industrial sectors, transportation, and agriculture in particular.^1^ Decreasing electricity feedstock prices can allow for the increased usage of electrochemically synthesized hydrogen, to both be competitive with conventional systems and to allow for the increased use of hydrogen in energy overall.^2,3^

Traditional alkaline electrolyzers (AELs) that use high concentration potassium or sodium hydroxide electrolytes have been operational commercially since 1927.^4^ However, due to the distance between electrodes in traditional systems, AELs operate at lower current densities and have a slower transient response, complicating the utilization of low cost and intermittent power. Proton exchange membrane (PEM) electrolyzers benefit from a solid electrolyte membrane and zero-gap configuration to reduce electronic resistance and allow for high current density operation with a faster transient response.^5–8^ However, the low pH and high potential environment at the anode limit the material choice to precious-metal catalysts and component coatings (separators and transport layers).^4^ Anion exchange membrane (AEM) electrolyzers can potentially combine the benefits of both AELs and PEM electrolyzers by making use of a hydroxide conducting polymer, which can allow for platinum group metal (PGM)-free electrocatalysts and component coatings (separators and transport layers). Moreover, the zero-gap solid electrolyte assembly also allows operation at high current densities and faster dynamic response.^2–4^

Over the past several years, there has been progress in the chemical stability of AEMs under alkaline conditions and the resulting electrolyzer performance of AEMs.^9,10^ Many of the reported cells operate with dilute KOH, although typically at a much lower concentration than that in AELs. With a KOH feed, factors limiting durability are different from those of pure water-fed AEM electrolyzers, and performance loss due to ionomer oxidation and catalyst layer detachment can be significantly delayed.^9,11^ Moreover, the presence of an electrolyte increases catalyst utilization beyond the catalyst layer-membrane interface, extending through the catalyst layer and well into the PTL structure. The improvements in AEM ionic conductivity and stability allow for extended operation and the examination of other individual components and degradation processes for their impact on the AEM cell performance and durability.

An understanding of porous transport layers (PTLs) and the roles that they fill can be leveraged in part from findings in PEM systems, where PTLs are critical in establishing electron transport pathways, promoting catalyst layer utilization, allowing for mass transport, and providing mechanical support to the polymer and catalyst layer.^12–19^ Of particular importance is that poor PTL-catalyst layer interfacial contact and increasing the in-plane distance for electron transport worsens catalyst layer utilization and catalyst layer resistances^20,17^ and in cases can worsen the high frequency resistance as well.^21,22^ For the improvement of PTL properties, the use of a microporous layer has been advantageous to improving interfacial contact and catalyst layer utilization, minimizing kinetic and catalyst layer resistance overpotentials.^23,24^ Laser ablation of PTLs has further been used to improve the interfacial properties of PTLs through a similar mechanism, improving cell kinetics and minimizing high frequency resistances.^25^ PGM coatings in PEM electrolysis are further critical to preventing passivation and performance losses through increasing high frequency resistance and lower catalyst layer utilization.^26–31^ In AEM systems, an additional complication is that the supporting electrolyte provides site access throughout the PTL,^13,32^ which creates materials challenges related to stability (passivation/dissolution) at operating pH/potential and where the PTL is a significant contributor to cell kinetics.^33^ PTLs for AEM electrolysis have generally focused on nickel (Ni) and stainless steel (SS)-based materials due to higher performance and relative stability, although both have durability concerns related to passivation (primarily Ni) and dissolution (SS).^34–37^ More recently, Hassan et al. investigated commercial materials, finding that the PTL composition and porosity/density were significantly more important for cell performance compared to the PTL thickness.^32^ Similar materials strategies have been used to improve interfacial contact and minimize high frequency and catalyst layer resistances, including microporous layers,^38,39^ PTL/catalyst interlayers,^40,41^ and cellulose nanofibrils.^42^ Additionally, AEM systems can leverage findings from AEL anode engineering and nanostructure development, as supporting electrolytes and the PTL site access blur the lines between these two electrolyzer technologies.^43–48^

MEA durability in AEM electrolyzers is evaluated, with a focus on commercial and unmodified PTLs and poly­(aryl piperidinium) (PAP) membranes.^49^ This study focuses on changing the anode PTL, and the possible impacts of PTL elements and properties may have on MEA performance and durability. Voltage breakdown analysis during test operation allows for a link between PTL materials/properties and the observed loss type (ohmic, kinetic, or residual), and the focus on longer-term operation helps address literature gaps. This work builds off of findings throughout the literature as well as past efforts screening a wider set of PTLs, both in material properties and composition, for AEM performance.^33,50^

## Experimental Section

### MEA Fabrication

Membrane electrode assemblies (MEAs) (25 cm^2^) were prepared as catalyst-coated membranes (CCMs) on the cathode by spraying directly onto 80 μm PiperIon AEM and catalyst-coated substrates on the anode by spraying onto the porous transport layer (PTL) material. Cathode catalyst layers contained Pt on high-surface-area carbon (Pt/HSC, Tanaka Kikinzoku Kogyo, TEC10E50E) with a Pt loading of 0.3 mg_Pt_/cm^2^ and an ionomer content of 30 wt %. Cathode catalyst layers were sprayed onto the 80 μm PiperIon AEM using an Accumist ultrasonic spray head in an automated spray station with an ink flow rate of 0.2 mL/min. Anode catalyst layers contained NiFeO_*x*_ (US Research Nano) with a loading of 2 mg/cm^2^ and an ionomer content of 22 wt %. Anode catalyst layers were sprayed onto the anode PTL using a Grex Tritium Double Action Gravity Airbrush, equipped with a 0.3 mm nozzle. The loadings were confirmed using X-ray fluorescence (XRF) spectroscopy, using a Fischer XDV-SDD energy-dispersive spectrometer (EDS) with 30 s exposure time, averaged over three measurements within the active area.

Two PTLs from Bekaert were used in MEA testing, Currento 2Ni 18–0.25 (Ni) and Bekipor XL601S AISI 316L (SS, Table 1). The Currento (Bekaert) PTL consisted of Ni with a thickness of 250 μm, a 60% porosity, and a fiber diameter of 20 μm. The Bekipor (Bekaert) PTL consisted of iron (65%), chromium (18%), Ni (14%), and molybdenum (3%) with a graded porosity, a thickness of 650 μm, a porosity of 79%, and fiber diameters of 38 μm (flow field interface) and 19 μm.

**Table 1:** Fiber Diameter, PTL Thickness, and Porosity of Ni and SS PTLs

Parameter	Ni PTL	SS PTL
Fiber diameter [μm]	20	38 ± 1.2, 19 ± 0.8
PTL thickness [μm]	250	650
Porosity [%]	60	79

While direct comparisons are preferable, they are not possible in the case of commercial Ni and SS PTLs. While SS is not a durable or suitable solution in AEM electrolysis, SS does allow for material properties not currently available in a Ni format. These properties include a graded material density/porosity (higher interfacial contact with the catalyst layer for kinetics and higher porosity at the flow field for transport), improved electron transport at operating potential, and smaller fiber-to-fiber distances. The comparison of the PTL materials with different properties was completed not to compare the viability of Ni versus SS but to demonstrate the possible advantages of translating the SS PTL properties to a Ni format.

### Electrochemical Testing and Characterization

Prior to testing, the CCM was soaked in 1 M potassium hydroxide (exchanged once after 24 h) for at least 48 h to convert the membrane and ionomer into the hydroxide form. The cell hardware was obtained from Fuel Cell Technologies with aluminum end plates and fitted with Ni flow fields. Ni tubing was welded in-house to the flow fields to prevent contact of the caustic electrolyte with the aluminum end plates. Potassium hydroxide was obtained from MilliporeSigma.

Cells were assembled using skived polytetrafluoroethylene (PTFE) gaskets to match the thickness of the anode electrode (11 MIL with the Ni PTL, 27 MIL with the SS PTL) and to set the compression of the carbon gas diffusion layer (GDL, AvCarb MGL280) to approximately 20% (9 MIL).

MEAs were tested at 80 °C in cathode dry mode and KOH flow recirculated to the anode at 50 mL min^–1^ on AEM degradation test stands at NREL. A P&ID diagram of AEM test station design is shown in Figure S1. The test station was built in house and electrolyte heating was completed in line prior to reaching the cell and through heating rods in the cell end plates. After passing through the cell, electrolytes were recirculated through a liquid/gas separator, sparged with nitrogen to prevent electrolyte carbonation. The separators were equipped with level sensors and water was added during the test to replace water from consumption (hydrogen generation) and carried by the sparging gas. There are advantages and disadvantages of each test approach (sparging gas, to add water or not, to add water in situ or periodically) that balance safety and test consistency, and this approach was used to ensure consistency in the electrolyte and applied stressor throughout durability testing. By adding water in situ, a momentary heterogeneity in the electrolyte concentration and temperature within the recirculation tanks was possible, although the water additions were gradual and extremely small. MEAs were further tested with a dry cathode, generally found to result in improved performance through a lower ohmic overpotential and slightly lower mass transport/residual loss (although higher kinetic losses).^50^ From multiscale modeling, it was believed that the hydroxide concentration gradient between anode/cathode was higher and improved mass transport and that feeding liquid electrolyte to the cathode may have further increased bubble entrapment.^50^

The polarization curves (PCs) were obtained anodically and cathodically under steady-state conditions by holding the cell at potentials between 1.4 and 2 V for 120 s and acquiring the current output. Electrochemical impedance spectroscopy (EIS) data were collected between 1.4 and 1.7 V using frequency ranges from 18 kHz to 1 Hz, which allowed for high frequency resistances (HFRs) to be reported. The durability was evaluated through a current hold of 1 A/cm^2^. In between the 1 A/cm^2^ holds, PCs, EIS measurements, and cyclic voltammograms between 0 and 1.3 V were taken repeatedly at regular intervals during the long durability test. Cell diagnostics and polarization curve analysis were used to examine the sources of the MEA performance loss. Ohmic/resistance loss was determined from the HFR and the difference between polarization curves uncorrected and those corrected for internal resistance. Transport/residual loss was determined from the difference between polarization curves corrected for internal resistance and the logarithmic relationship between the cell voltage and current.

Station shutdowns occurred throughout durability testing at 1 A/cm^2^, 11 times in 1000 h for the Ni PTL and 10 times in 1400 h for the SS PTL. The majority of these shutdowns were planned (8 for both the Ni and SS PTL) to monitor changes to the polarization curves, diagnostics, and loss type; on occasion, station shutdowns were unplanned (3 for the Ni PTL and 2 for the SS PTL) and related to experiment-safety considerations (loss of sparging gas during bottle changes), laboratory shutdowns, and electrical loss. Tests were immediately resumed and were not ramped in any way, and differences in voltage response were related to composition difference (Ni, SS) in the anode PTL. Prior to and following this study, MEAs with the same membrane and cathode (Pt/HSC cathode) have been found to have significantly lower, no, or negative loss rates. The most significant factors in the voltage loss rate have been found to be related to the anode catalyst layer and PTL composition, as well as electrolyte concentration and the magnitude of the applied stressor.^50^

Broadly, two-electrode MEA data are limited in several ways. Kinetics combine the anode and cathode (particularly in alkaline systems with a larger cathode contribution) and contributions from the anode catalyst layer and anode transport layer in supporting electrolytes. There are also aspects of materials integration (electrode fabrication, ionomer content) that clearly alter catalyst site access, reactivity, and cell performance beyond intrinsic catalyst activities. Throughout any electrochemical system, there can further be a disconnect between steady-state operation and polarization curves/diagnostics due to differences in the applied current/potential and the redox potentials from different components. Studies leveraging MEA testing, however, are critical in building a fundamental understanding of materials to the demonstration and optimization of an applied technology.

### Physical Characterization

To measure through-plane resistance, also termed interfacial contact resistance (ICR), the PTL was sandwiched between gold-coated plates under a defined compression. A four-wire resistance measurement with DC current was used, and the plate-to-plate voltage drop was measured to calculate the ICR as a function of compression. ICR and HFR measurements are related but are not the same primarily because ICR measurements are taken between two rigid gold plates and without a compressible (cathode transport layer) or flexible (membrane) component to improve interfacial contact. While ICR increases may result in an HFR increase, they may also negatively impact kinetics due to the lower oxygen evolution reactivity of oxides.^51^ Additionally, the HFR is largely dictated by the membrane thickness/chemistry, and the impact of anode passivation on the HFR may be negated by the membrane contribution and partially mitigated by the supporting electrolyte. During cell testing, a cathode compression of 20% would result in a force of 0.13 MPa on the anode. This value is below the scale of an ICR measurement (0.5 MPa minimum), and in this way, ICR values are more a qualitative guide as to the possible causes of HFR/kinetic changes and do not provide a clean delineation of HFR contributions.

The surface morphology of PTLs was investigated using a Hitachi S-4800 field emission scanning electron microscope at an accelerating voltage of 2–10 kV. The samples (bare, pretest coated, and post-test coated) were dried in air before the scanning electron microscopy (SEM) analysis and attached on the holder using copper tape. Only the top-down images were obtained. SEM was also used for elemental analysis using energy-dispersive X-ray spectroscopy (EDS) tool at an accelerating voltage of 15 kV.

Scanning transmission electron microscopy (STEM) was performed using a JEOL NEOARM analytical electron microscope operated at 200 kV and equipped with dual windowless silicon-drift detectors each with a 100 mm^2^ active area. Specimens were prepared by scrapping off the catalyst from the PTL and dispersed in isopropanol alcohol followed by drop-casting onto the TEM grid. High-angle annular dark-field (HAADF) images and EDS maps of catalyst agglomerates were recorded. EDS data was processed with the Analysis Station software (JEOL Ltd.). Electron energy-loss spectroscopy (EELS) was also recorded to examine any change in the electronic state of the catalyst. A custom Python code was used to analyze EELS data. EDS can be surface-sensitive, and compositional changes can be influenced by statistics (the small collection area during EDS), the electrolyte, and the presence of carbon. While the higher mobility of iron relative to nickel may not be established through EDS alone, the higher mobility of iron is expected throughout literature and other studies with the same materials leveraging inductively coupled plasma mass spectrometry.^50–54^

## Results and Discussion

### Origin of Cell Performance Differences with PTLs

SS and Ni PTLs were used as part of MEAs and their impact on cell performance and durability was investigated. Table 1 includes properties for the two PTLs utilized in this study, SS and Ni. The SS PTL has a graded porosity, where the sides facing the membrane and flow fields had fiber diameters of 19 and 38 μm, respectively. For both PTLs, the fiber diameter facing the membrane was similar and the primary differences were in thickness (SS thicker) and porosity (SS bulk more porous).

Figure 1a compares the initial performance of MEAs with PAP membranes and ionomers fed with 1 M KOH on the anode side using the Ni and SS PTL materials at the anode. The difference in performance was significant, and the current density was 730 mA/cm^2^ higher at 2 V (2.92 A/cm^2^ for SS PTL and 2.19 A/cm^2^ for Ni PTL at 2 V) and 390 mA/cm^2^ higher at 1.8 V (1.39 A/cm^2^ for SS PTL and 1.0 A/cm^2^ for Ni PTL at 1.8 V) for the MEA with SS PTL compared to that of the MEA with Ni PTL. Further examination of the HFR-free voltages in Figure 1b demonstrates differences in the low-current-density performance between the Ni and SS PTLs. While some of this difference may be due to the PTL elemental composition impacting the kinetic mechanism, the increasing deviation and higher Ni voltage at moderate current density suggest possible issues in a catalyst layer resistance or mass transport, further reflected in the higher residual loss in Figure 1c.

**Figure 1:**
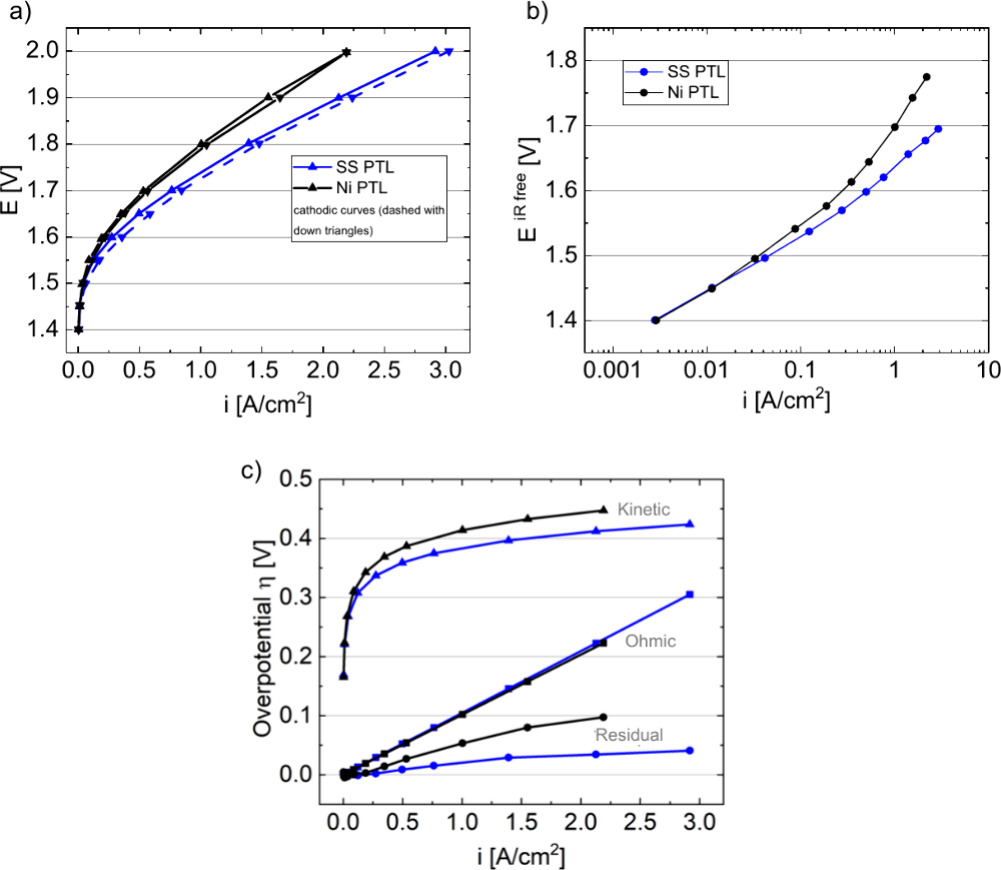
(a) Polarization curve; (b) logarithmic kinetic plot and (c) breakdown of kinetic, ohmic, and residual losses collected at 80 °C in 1 M KOH, Pt/HSC sprayed on 80 μm PiperION membrane as cathode (Pt: 0.3 mg/cm^2^) and NiFeO_*x*_ sprayed on Ni PTL (black) and SS PTL (blue) (NiFeO_*x*_ ∼2 mg/cm^2^).

The residual overpotentials included all overpotentials that were not Ohmic (separated with high-frequency-resistance measurements) or kinetic (separated through logarithmic or Tafel analyses of polarization curves). Residual losses include mass transport (water and oxygen/hydrogen bubble transport) as well as electronic (not accounted for in the high frequency resistance) and ionic resistances that limit site utilization at higher currents. For the residual losses in Figure 1c, overpotential differences are primarily due to material differences in the anode (catalyst/transport layer combined). Additionally, these losses are generally larger than for standard material sets in proton exchange membrane systems.^20,55^ Other studies have hypothesized that much of this difference in residual loss may be due to differences in the in-plane resistance, where an overpotential penalty is taken due to the distance electrons travel through the catalyst layer to reach transport layer fibers.^50^ The Ni transport layers may produce higher in-plane electronic resistances due to the tendency of Ni to passivate at elevated pH and potential, limiting electron transport during operation. The SS transport layer may further minimize electronic resistances due to the higher material density at the catalyst layer interface, reducing the distance for electron transport. Additional differences in ionic transport are less likely due to consistencies in the catalyst layer, ionomer content, and support electrolyte used during testing.^55^ Compared with the Ni transport layer, the SS has a higher average porosity that may aid mass transport, although greater material density (lower porosity) at the catalyst layer interface may lessen or complicate any mass transport benefit. Logarithmic kinetic plots are complicated in MEA testing, combining anode/cathode kinetics, site access and reactivity, catalyst activity, and electrode integration challenges into a single response. While these types of plots cannot be used to assess mechanism or reactivity fundamentals, there is value in these types of plots in separating nonidealities in performance and establishing loss breakdown analyses at the cell level (Figure S2a,b).

Compared to the SS PTL (Figure S5b), the Ni PTL (Figure S5a) had a much lower ICR when measured ex situ. This may be due to a lower oxide content for Ni PTL compared to SS PTL, as suggested by SEM EDS analysis for the bare PTLs (Figures S3 and S4 and Tables S1 and S2). Much of this difference, however, may be negated following conditioning prior to the acquisition of electrochemical performance data (polarization curves and impedance). The difference in ICR can also be affected by the difference in thickness and porosity, where the Ni PTL was 250 μm thick with lower overall porosity 60% compared to 650 μm thick SS PTL with 79% porosity. However, the HFR, which includes electronic contact resistances (PTL/membrane and PTL/flow field interface),^56^ is comparable in situ with both PTLs (Figure 1c). This discrepancy, a large difference in ICRs and similar HFRs, is likely affected by the large membrane contribution to the HFR. The differences in ICR values may also be accentuated by how the ICR measurements are taken, between two rigid gold plates and without a compressible (cathode transport layer) or flexible (membrane) component to improve interfacial contact, and further do not account for resistance changes during conditioning prior to the impedance acquisition. HFR similarities may have further been due to the presence of the supporting electrolyte, which allows greater access of material through the pores of the PTL and may compensate for differences in the interfacial contact area of the PTL with the catalyst layer and flow field.

Significant differences, however, were seen in the kinetic and residual overpotentials, where the SS PTL resulted in much lower values for both. Even though the catalyst loading and type was similar for both tests, the lower kinetic overpotential with the SS PTL was likely due to better catalyst utilization, particularly at higher current densities. It has previously been shown by Xiao et al. and Kreider et al. that PTL materials offer catalytically active surface species throughout the pores of the PTL resulting in an increased site access;^4,33^ since 1 M KOH was used in testing, the sites within the PTL were likely OER-active. Since the SS PTL was thicker compared to the Ni PTL, it may further have offered more catalytic active sites resulting in an increased kinetic performance. The presence and anticipated mobility of iron may further improve the anode OER reactivity through iron incorporation into oxyhydroxide species or the improvement of electron transport.^50–54,57^ Moreover, the MEA with the SS PTL exhibited lower residual losses. This may be due to a smaller catalyst layer resistance and differences in electron transport due to a lower oxide content and higher elemental mobility during operation. The lower residual loss may also be due to differences in mass transport, a higher PTL porosity, and larger fiber distances further from the catalyst layer interface.

### Voltage Change with 1 A/cm^2^ i-Hold

The stability of both cells was evaluated for >1000 h of operation at 1 A/cm^2^ as shown in Figure 2a for the Ni PTL and Figure 2b for the SS PTL. Overall, both cells demonstrated degradation rates of 144 and 115 μV/h over 1000 h of operation for the cells with Ni and SS PTLs, respectively. These loss rates, however, are higher than found in other cases, and studies with the same cathode (Pt/HSC, CCM) and GDL, membrane/thickness, and Ni PTL have since found far lower degradation rates when avoiding iron in the anode catalyst layer.^50^ The reported degradation rate values were taken at approximately 0 and 1000 h, before a KOH change and diagnostics. For the SS PTL shown in Figure 2b, the experiment was allowed to run longer due to a lower cell potential after 1000 h of testing. The degradation rate increased rapidly after 1290 h, however, and the test was stopped after the cell voltage exceeded 2.2 V.

**Figure 2:**
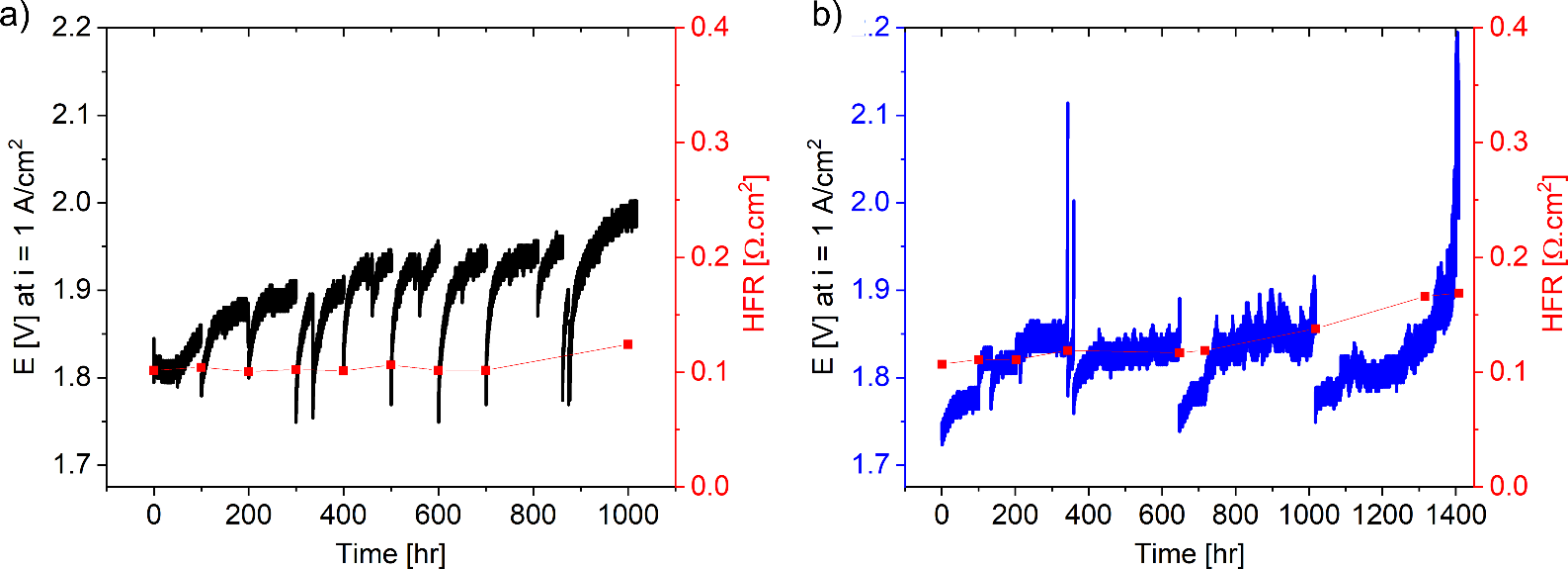
Cell voltage and HFR changes over time, with MEAs using a (a) Ni and (b) SS PTL during durability testing at 1 A/cm^2^.

It is also important to note that KOH concentration may increase over time due to water consumption and evaporation from the recirculation bottles as the feed to the cell was heated to 80 °C and is discussed in greater detail in the Experimental Section. To avoid concentration changes, level sensors were put in place, which would trigger the flow of DI water into the bottles and were critical, since differences in the KOH concentration can significantly influence the ionic conductivity, polymer stability, and the electrode activities in the hydrogen evolution (HER) and oxygen evolution (OER) reactions.^58^

In all instances, stopping cell operation for diagnostics, an unplanned shutdown, or a change in the KOH electrolyte resulted in an improvement in performance when the test was restarted. The voltage decreases (improvements) at various instances as observed in Figure 2a,b were likely due to a combination of partial anode reduction to a lower redox state (kinetics, secondarily catalyst layer resistance and ohmic) and the clearing of trapped gas bubbles (mass transport and kinetics). The degree of voltage improvement was consistently larger for the Ni PTL compared with SS. This may have been due to mass transport (porosity differences) or differences in elemental mobility that can affect the in-plane electronic resistance (catalyst layer resistance).^55^ It was also possible that taking cyclic voltammograms during diagnostics could have further exacerbated partial reduction of the anode catalyst and PTL, resulting in a greater improvement in the cell voltage.

The average HFR for the cells was taken at various times during the test and plotted with the durability results in Figure 2a,b. Initially, the HFR was very similar whether using Ni PTL (102 mΩ·cm^2^) or SS PTL (107 mΩ·cm^2^), indicating that the ex situ ICR measurement was not fully indicative of the HFR measurement in situ. Post-test, the ICR increased more drastically for the Ni PTL (although it was still lower than the SS PTL), and much of this increase may have occurred during conditioning and prior to performance/durability testing. It is important to note that for the SS PTL, there was a gradual increase in the HFR (57.8% increase at 1400 h; 28.7% increase after 1000 h) that at the end of test was higher than the test that used the Ni PTL (22.6% increase at 1000 h). The HFR increases at 1000 h were similar, however, although the exposure to >2 V for the SS PTL may have caused greater irreversible loss due to corrosion compared to Ni PTL passivation.

### Voltage Breakdown over Long-Term Durability

Polarization curves and EIS measurements were taken at intervals throughout the durability test, which allowed for a voltage breakdown into ohmic, kinetic, and residual parts. The voltage breakdown associated with residual losses may have included losses from the catalyst layer resistance through electronic/ionic transport limiting catalytic site access at higher current densities.^20^ It may also have included mass transport losses due to changes in PTL pore structure, as well as ionomer/catalyst and catalyst layer movement and property changes.

Figure 3 shows the polarization curves (Figure 3a) and breakdown of Ohmic (Figure 3b), kinetic (Figure 3c), and residual (Figure 3d) overpotentials as a function of current density for the Ni PTL cell. There was minimal change in the ohmic overpotential during the first 700 h (Figure 3b). In the last 300 h before EOT, there was an increase in the ohmic overpotential by 22 mV at 1 A/cm^2^. In comparison to the ohmic loss, however, there was a larger increase in the kinetic overpotential, 40 mV at 1 A/cm^2^ from BOT to EOT (Figure 3c and Figure S2a). Similarly, the residual overpotential increased with time, approximately 87 mV at 1 A/cm^2^, with most of this increase occurring during the first 500 h (Figure 3d).

**Figure 3:**
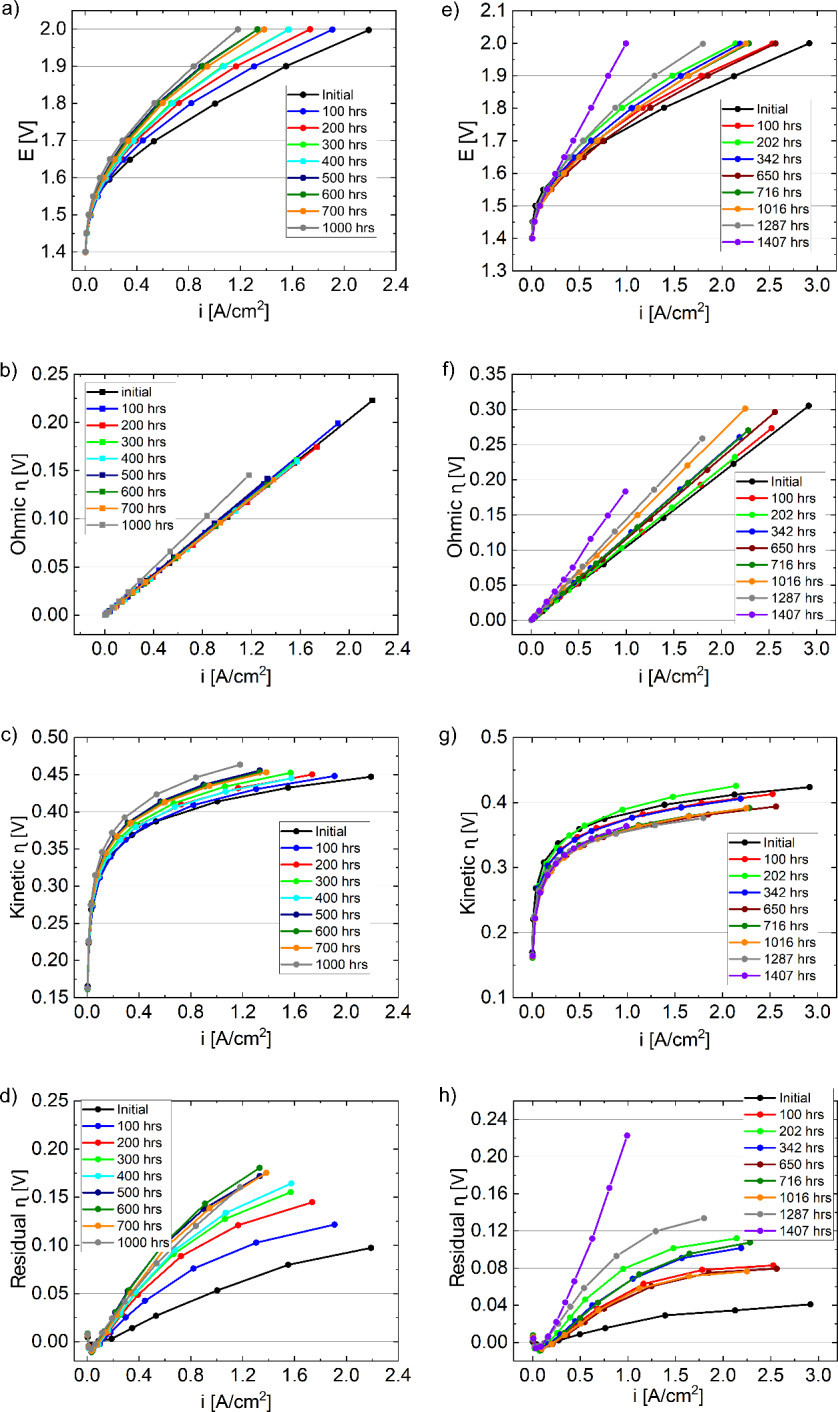
Durability data of MEAs with a (a–d) Ni PTL and a (e–h) SS PTL at the anode. Data includes (a, e) polarization curves and breakdowns of (b, f) ohmic, (c, g) kinetic, and (d, h) residual losses over time. Polarization curves and EIS measurements were taken periodically over the course of durability testing at 1 A/cm^2^.

The relatively small increase in the Ohmic overpotential may be due to several possible reasons (Figure 3b). At high anodic potentials, there was a possibility of Ni PTL passivation due to oxidation, which was suggested by EDS (Table S1) where the oxygen content on the Ni fiber increased compared to pretest Ni PTL (sprayed) and bare Ni PTL. Post-test ICR results shown in Figure S5 also suggest that there was an increase in the interfacial contact resistance. Additionally, changes in the membrane could also cause an increase in the Ohmic overpotential. These impacts, however, were likely small since the increase in ohmic overpotential was only 22 mV at 1 A/cm^2^ for the test duration. Much of the PTL oxidation (EDS in Table S1 and ICR in Figure S5) may have occurred during conditioning, minimizing the impact on durability testing.

For the Ni PTL MEA, the increase in kinetic overpotential was more significant and likely due to a combination of factors (Figure 3c and Figure S2a). Passivation and oxide growth within both the catalyst layer and PTL may have resulted in a lower OER reactivity.^33,59,60^ The NiFeO_*x*_ catalysts pre- and post-test, however, show similar structures which resemble that of a spinel (in [211] zone axis) with (111) planes and *d*-spacing about 0.49 nm (Figure 4a–c). EELS analysis further showed no clear change in the oxygen, iron, and Ni peak locations during testing, suggesting similarities in the iron and Ni oxidation states within the catalyst layer (Figure 4d–f). If losses due to passivation and oxide growth occurred, they likely occurred predominantly in the PTL and may speak to the high degree of PTL participation to OER and cell kinetics.^33^ Additionally, EDS suggested an expected iron loss from the catalyst layer (Tables S1 and S3), where Fe:Ni atomic ratio changed from 2 to 3:1 (depending on energy, area evaluated) to almost 1:1. While the initial iron content was likely too high for optimal catalyst reactivity, a possible but anticipated iron mobility during testing may have further segregated iron/Ni zones and limited the iron benefit to Ni-OER activity.^61–63^ Some catalyst layer delamination was also observed visually as a coloration of the electrolyte in the recirculation tanks and likely resulted in lower site quantity. Top-down SEM images of the Ni PTL pre- and post-tests (Figure 5) also showed catalyst layer movement into the PTL pores and delamination, which could contribute to kinetic loss as well as ohmic loss through an increased contact resistance. Microscopy of the catalyst layer removed from the PTL before and after testing (Figure 4a–c) showed small changes in the catalyst aggregate size, although some agglomeration was possible and could have accounted for a loss in site access. Additionally, high-resolution STEM indicated minimal changes to the catalyst lattice, by evaluating the (111) and (220) distances before and after testing, and suggested that alloying/dealloying processes were not a primary contributor to changing reactivity.

**Figure 4:**
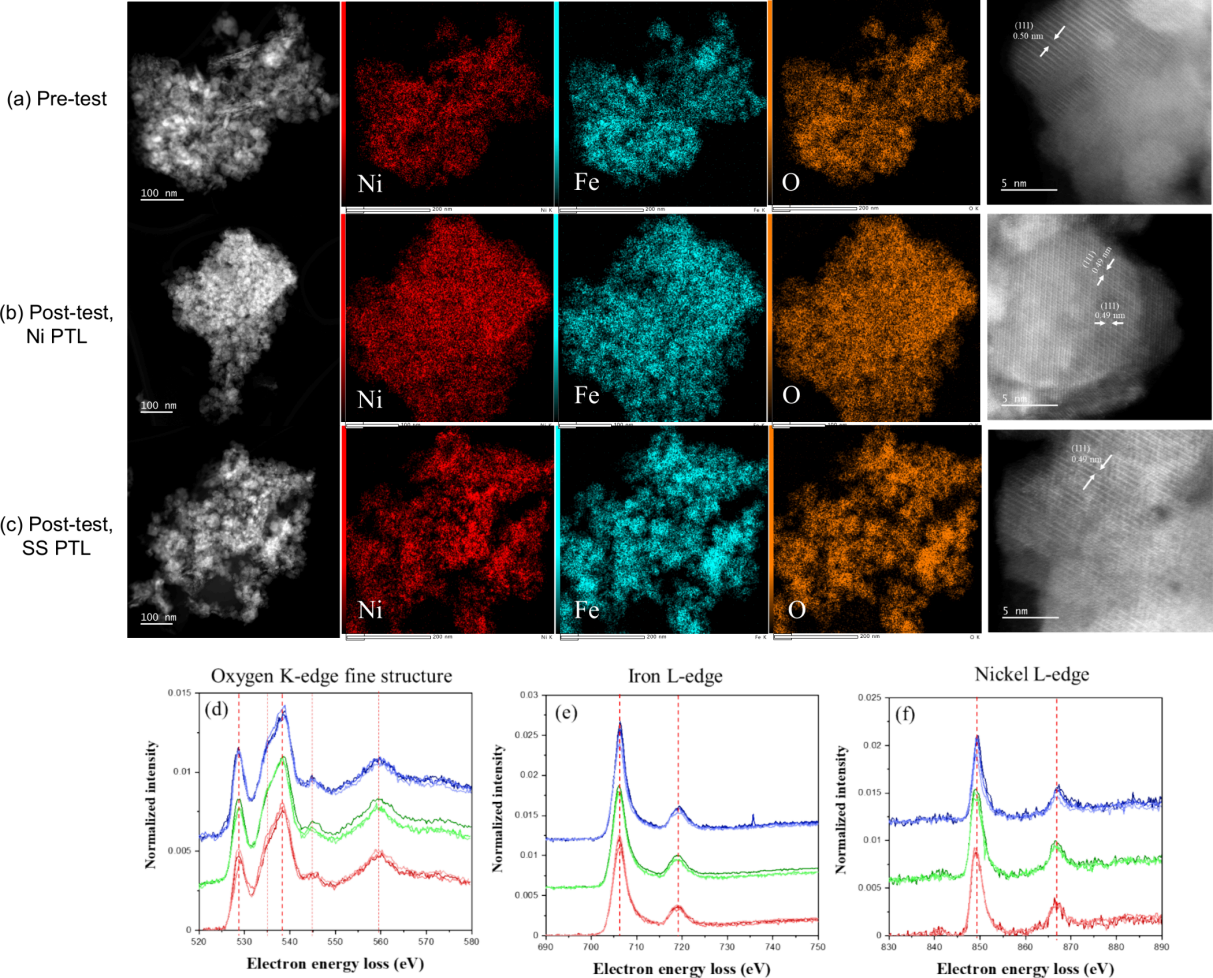
STEM of the anode catalyst layer (NiFeO_*x*_) (a) pretest and post-test with a (b) Ni PTL and (c) SS PTL. HAADF images and EDS maps were included for Ni (red), iron (blue), and orange (oxygen) signals. EELS analysis of the (d) oxygen K-edge, (e) iron L-edge, and (f) Ni L-edge of the anode catalyst layer (NiFeO_*x*_) pretest (red from SS PTL) and post-test (blue from Ni PTL, green from SS PTL).

**Figure 5:**
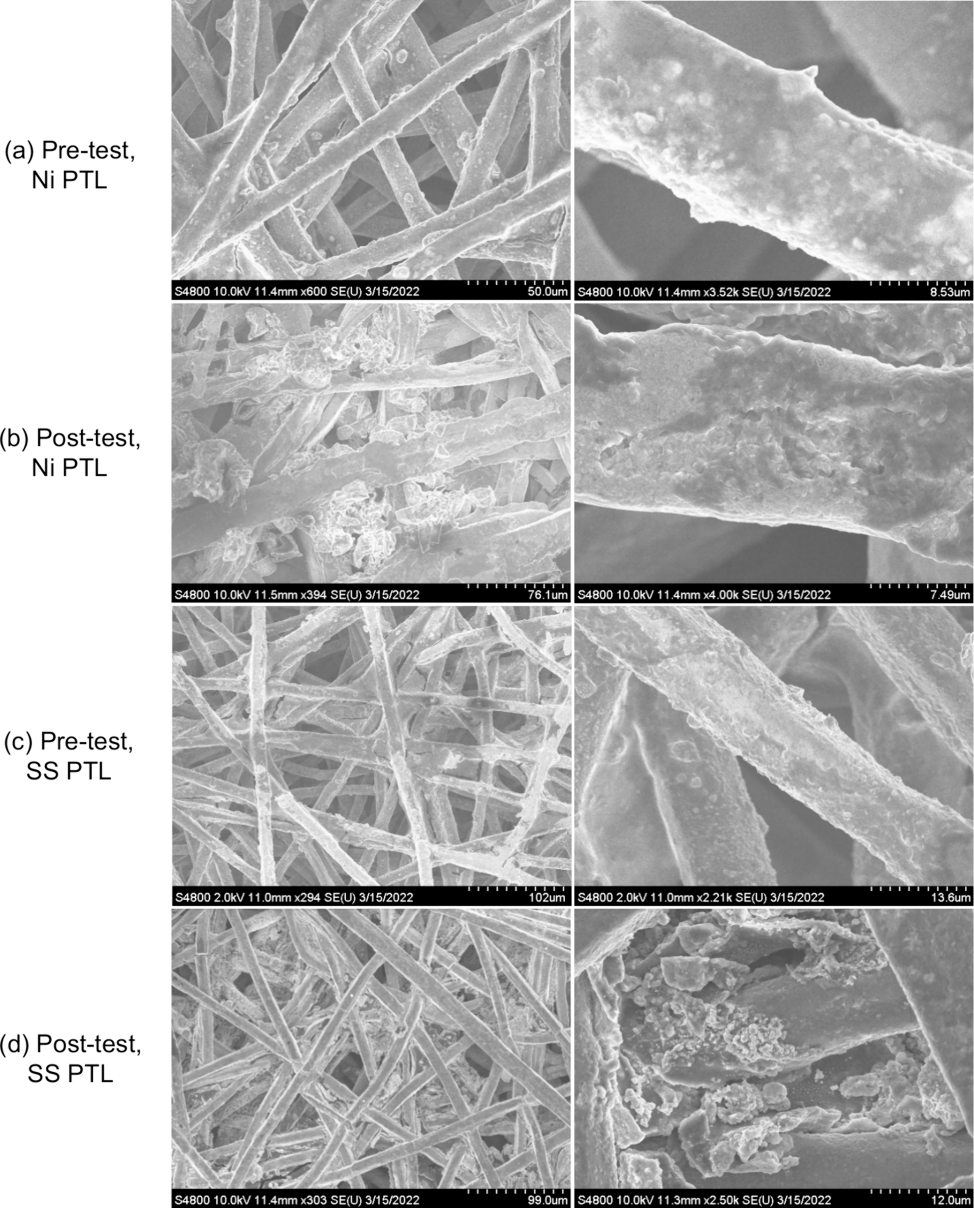
SEM images of (a, b) Ni PTL and (c, d) SS PTL sprayed with NiFeO_*x*_ catalyst pretest and postdurability test (1000 h i-hold at 1 A/cm^2^).

The residual overpotential increase was also significant (Figure 3d). Catalyst layer resistances may originate from electronic and ionic transport limitations in the catalyst layers. Compared with the SS PTL, the Ni PTL residual losses were higher and may indicate an in-plane electronic resistance from the higher porosity at the catalyst layer interface.^55^ Due to the anode catalyst loading (2 mg/cm^2^), ionomer content (22 wt %), and electrolyte concentration (1 M KOH), through-plane electronic and ionic transport limitations were likely less impactful.^55^ Increasing residual losses could also have been due in part to ionomer degradation and partial catalyst layer delamination resulting in lower in-plane electronic transport, as well as ionic transport limitations due to ionomer loss. Additionally, mass transport may contribute as well, and post-test images of the Ni PTL show some pores blocked with the delaminated catalyst layer, resulting in a change in pore structure.

Voltage breakdown analysis was also completed for the cell with an SS PTL. Figure 3 shows the polarization curves (Figure 3e) and a breakdown of ohmic (Figure 3f), kinetic (Figure 3g), and residual (Figure 3h) overpotentials as a function of current density. In contrast to the Ni PTL, the SS PTL exhibited a gradual increase in the Ohmic overpotential throughout the test duration, amounting to 79 mV at 1 A/cm^2^ by the EOT (Figure 3f). While there was some variability in the kinetic overpotential and an increase between BOT and 200 h, the kinetic overpotential generally decreased and by EOT was near the optimum and approximately 20 mV lower than the BOT (Figure 3g and Figure S2b). A significant change in the residual overpotential was further observed, which increased by 202 mV at 1 A/cm^2^ (Figure 3h).

Possible reasons behind the increase in the ohmic overpotential include passivation, ion blocking within the polymer and cathode contamination, chemical degradation of the polymer, and anode dissolution and contact loss. Increases in the ohmic overpotential can be due to catalyst/PTL passivation and oxide growth, EDS (CCS, catalyst/PTL) suggested qualitative increases in the oxide content (Table S2), and ICR measurements showed an increase in the contact resistance (Figure S2b). EDS (Figure 4a–c) and EELS (Figure 4d–f) of the catalyst layer, however, suggested similarities in the catalyst (iron and Ni) oxidation state after testing. The anticipated higher mobility of iron can further ion block within the membrane and adversely impact the cathode. Cation contamination, however, is expected in all tests with a supporting electrolyte, including a ppm level of iron and Ni content.^64^ Additionally, iron is expected to improve oxygen evolution and hydrogen evolution reactivity, although a negative impact is expected with a platinum-based cathode.^61,65–68^ Supporting evidence of a particularly negative contamination effect was not found, including EDS of the membrane/cathode, a changing shape or hysteresis in the polarization curve, or a low frequency feature in impedance.^69^ Chemical degradation of the polymer could also contribute to HFR changes, although this was not supported in post-test characterization or in the polarization curve where a short could be possible. Anode PTL dissolution may further contribute to worsening electron transport. Although not directly confirmed in this study, the possibility of increased iron mobility is suggested by EDS (Table S2) and expected from literature and other studies.^50–54^ It was also was accompanied by an expected increase in residual loss (catalyst layer resistance), suggesting changes (loss) to the interfacial contact.^55^

Contrary to what was seen with the Ni PTL, the kinetic overpotential with the SS PTL generally decreased with time during the test (Figure 3c,g and Figure S2a,b) and was potentially due to the higher degree of iron mobility at the pH and potentials during testing. While speculative and not directly confirmed here, the broad mobility of iron/chromium is supported throughout literature and other studies on these materials.^50–54^ Materials changes, however, were observed and included delamination of the catalyst layer into the PTL pores, as found in top-down SEM images before and after testing (Figure 5). While catalyst delamination was generally expected to worsen kinetics, other studies have found higher performance with a bare SS PTL than with a catalyst layer included.^33^ Additionally, it was also possible that the graded pores of the SS PTL trapped more of the delaminated catalyst layer compared to the Ni PTL, preserving catalyst sites. Although the porosity of SS PTL was higher throughout the bulk, the higher material density at the catalyst layer interface likely aided with catalyst entrapment and was previously found to limit catalyst layer delamination in AEM electrolyzers with water-only feeds.^50^

During initial testing, the SS PTL residual losses were less than that of the Ni PTL (Figure 3d,h) and may have been due to the graded porosity and greater interfacial contact with the catalyst layer, reducing in-plane electronic resistances. Following durability testing, however, the increase (202 mV at 1 A/cm^2^) was larger than that for the Ni PTL. This may have been due to the increased mobility of iron, potentially resulting in larger through-plane electronic (increased PTL porosity and increased catalyst layer delamination), in-plane electronic (increased dissolution/loss of PTL), and ionic (increased catalyst layer delamination) limitations. Additionally, catalyst delamination and entrapment within the PTL pores may have caused a change in the pore structure, increasing mass transport limitations.

In relatively short-term testing, including the durability tests completed here, the iron content in the PTL may benefit cell kinetics and electronic conductivity within the electrode due to its anticipated higher mobility by distributing small amounts of iron throughout the electrode and replacing the iron otherwise lost within the catalyst layer during operation. Long-term, however, iron is thermodynamically favored to dissolve at this pH and these potentials, and with significantly longer testing, iron may lead to additional degradation drivers, including: declining reactivity due to the loss of from the catalyst and transport layers; increasing electronic resistance (ohmic and residual, observed in this study) due to the erosion of material from the transport layer; and possible degradation of the polymer (or higher degrees of ion blocking within the polymer).

## Conclusions

With recent improvements in the stability and ionic conductivity of membranes, other component choices and their integration into MEAs are critical in establishing and improving upon cell performance and durability. The anode porous transport layer (PTL) plays an essential role in AEMWE, allowing for the transport of electrolyte and products, as well as providing oxygen evolution reactivity, electron pathways, and a mechanically supportive substrate for the catalyst layer/membrane. This study focuses on Ni- and SS-based PTLs to demonstrate the status of commercially available Ni PTLs and the possible cell efficiency/durability differences found with MEAs using PTLs containing other elements (iron/chromium) and material properties. While cell voltages and their changes over time are clearly based on several components and their integration/interfaces, a few conclusions can be drawn from this study:

- The initial performance was higher with the SS than the Ni PTL, due to lower kinetic and residual losses. This was possibly due to differences in PTL density and interfacial contact with the catalyst layer, affecting in-plane electronic and ionic transport (less catalyst layer segmentation) as well as higher porosity in the bulk possibly improving mass transport.
- Relatively short-term durability (>1000 h) at 1 A/cm^2^ was demonstrated, achieving degradation rates of 144 μV/h and 115 μV/h over 1000 h of operation for Ni PTL and SS PTL respectively.
- Voltage breakdown analysis confirmed that an increase in the residual overpotential was the most significant cause of degradation for tests with both the Ni PTL and SS PTL. Increasing residual losses may have been due to catalyst layer loss (polymer degradation and partial catalyst layer delamination), PTL passivation/dissolution, and PTL structural changes that could have affected electronic, ionic, and mass transport pathways.
- Voltage fluctuations were observed during planned and unplanned station shutdowns, likely due to reversible passivation losses and operational stoppage clearing of entrapped bubbles. It may be critical to separate passivation losses for greater accuracy when defining an irreversible loss rate.
- SS PTL properties may have contributed to a higher retention of kinetics in short-term durability tests, including the iron content and mobility improving catalyst reactivity and the lower porosity trapping delaminated catalysts within the transport layer.

Ni PTLs are often employed in AEM anodes due to their long-term stability at high pH and potential. Commercial Ni materials, however, have largely not been optimized for AEM electrolysis (often coming from separation processes and batteries) and may be lacking in terms of large fiber distances hindering interfacial contact, mechanical reinforcement of the membrane, and catalyst layer retention during ionomer oxidation and/or catalyst dissolution. The elements within SS are not thermodynamically stable at high pH/potential, and the SS PTLs are likely not an acceptable long-term solution for electrolysis, possibly demonstrated by the SS PTL cell failure at 1400 h and the elevated degradation rates in both MEAs due to the iron content within the catalyst. SS, however, was useful in this case since the material properties (porosity, grading, fiber distance, interfacial contact, and improved electron transport) may potentially benefit AEM electrolysis.

PTLs in AEM electrolysis are an underexplored area relative to PEM systems. While much understanding can be gleaned from PEM/AEL developments, many of these materials may not be viable in AEM electrolysis due to the supporting electrolyte and PTL coating site access, and a need exists to better understand the impact that material properties (porosity/density, fiber distance, and interfacial contact) have on cell efficiency and durability within AEM systems. Broadly, understanding the impact of transport layers on the cost, efficiency, and lifetime is critical to the advancement of electrochemical systems, particularly in supporting electrolytes that increase access and utilization to these components.

## Supplementary Material



## Notes

The Supporting Information section contains: The Supporting Information is available free of charge at https://pubs.acs.org/doi/10.1021/acsomega.6c01506.

A P&ID of the AEM test station; ICR data of the electrodes; MEA kinetic data; microscopy and EDS data of the electrodes before and after testing (PDF)

## #

Honeywell, 201 W Oakton Street, Suite C, Des Plaines, Illinois 60018, United States of America

## $

Mott Corporation, 84 Spring Lane, Farmington, Connecticut 06032, United States of America

## References

[1] Miller H. A., Bouzek K., Hnat J., Loos S., Bernäcker C. I., Weißgärber T., Röntzsch L., Meier-Haack J. (2020). Green hydrogen from anion exchange membrane water electrolysis:
a review of recent developments in critical materials and operating
conditions. Sustainable Energy & Fuels.

[2] Alia S. M., Ha M.-A., Ngo C., Anderson G. C., Ghoshal S., Pylypenko S. (2020). Platinum–nickel
nanowires with improved hydrogen
evolution performance in anion exchange membrane-based electrolysis. ACS catalysis.

[3] Ghoshal S., Pivovar B. S., Alia S. M. (2021). Evaluating
the effect of membrane-ionomer
combinations and supporting electrolytes on the performance of cobalt
nanoparticle anodes in anion exchange membrane electrolyzers. J. Power Sources.

[4] Xiao J., Oliveira A. M., Wang L., Zhao Y., Wang T., Wang J., Setzler B. P., Yan Y. (2021). Water-fed hydroxide
exchange membrane electrolyzer enabled by a fluoride-incorporated
nickel–iron oxyhydroxide oxygen evolution electrode. ACS Catal..

[5] Chatterjee S., Peng X., Intikhab S., Zeng G., Kariuki N. N., Myers D. J., Danilovic N., Snyder J. (2021). Nanoporous iridium
nanosheets for polymer electrolyte membrane electrolysis. Adv. Energy Mater..

[6] Tan A., Song J., Qiu X., Liu Z., Xia L., Ju C., Zhao F., Li G., Shi X., Li T. (2025). Catastrophic localized deflagration formation mechanisms and safety
precautions in proton exchange membrane water electrolyzer. Nat. Commun..

[7] Tan A., Zhao F., Zhang Y., Li G., Wu C., Liu Z., Li J., Liu J. (2025). Innovative application of transfer
learning on small-scale datasets: Analysis and optimization of catalyst
ink for the low-iridium membrane electrode assemblies of proton exchange
membrane water electrolysis. Chem. Eng. Sci..

[8] Liu C., Ju C., Ma Y., Yao C., Zhang X., Liu Y., Zhang K., Li X., Tan A., Liu J. (2026). Spatially
defined and ultra-thin Pt coatings via interface engineering for cost-effective
proton exchange membrane water electrolysis. J. Power Sources.

[9] Hassan N. U., Mandal M., Zulevi B., Kohl P. A., Mustain W. E. (2022). Understanding
and improving anode performance in an alkaline membrane electrolyzer
using statistical design of experiments. Electrochimica
acta.

[10] Huang G., Mandal M., Hassan N. U., Groenhout K., Dobbs A., Mustain W. E., Kohl P. A. (2021). Ionomer
optimization
for water uptake and swelling in anion exchange membrane electrolyzer:
hydrogen evolution electrode. J. Electrochem.
Soc..

[11] Hassan N. U., Zheng Y., Kohl P. A., Mustain W. E. (2022). KOH vs
deionized
water operation in anion exchange membrane electrolyzers. J. Electrochem. Soc..

[12] Yuan X.-Z., Shaigan N., Song C., Aujla M., Neburchilov V., Kwan J. T. H., Wilkinson D. P., Bazylak A., Fatih K. (2022). The porous
transport layer in proton exchange membrane water electrolysis: perspectives
on a complex component. Sustainable Energy &
Fuels.

[13] Kang Z., Alia S. M., Young J. L., Bender G. (2020). Effects of various
parameters of different porous transport layers in proton exchange
membrane water electrolysis. Electrochim. Acta.

[14] Grigoriev S., Millet P., Volobuev S., Fateev V. (2009). Optimization of porous
current collectors for PEM water electrolysers. International journal of hydrogen energy.

[15] Kang Z., Mo J., Yang G., Retterer S. T., Cullen D. A., Toops T. J., Green J. B., Mench M. M., Zhang F.-Y. (2017). Investigation
of thin/well-tunable liquid/gas diffusion layers exhibiting superior
multifunctional performance in low-temperature electrolytic water
splitting. Energy Environ. Sci..

[16] Schuler T., De Bruycker R., Schmidt T. J., Büchi F. N. (2019). Polymer
electrolyte water electrolysis: correlating porous transport layer
structural properties and performance: Part I. Tomographic analysis
of morphology and topology. J. Electrochem.
Soc..

[17] Schuler T., Schmidt T. J., Büchi F. N. (2019). Polymer electrolyte water electrolysis:
correlating performance and porous transport layer structure: Part
II. Electrochemical performance analysis. J.
Electrochem. Soc..

[18] Zlobinski M., Schuler T., Büchi F. N., Schmidt T. J., Boillat P. (2020). Transient
and steady state two-phase flow in anodic porous transport layer of
proton exchange membrane water electrolyzer. J. Electrochem. Soc..

[19] Doan T. L., Lee H. E., Shah S. S. H., Kim M., Kim C.-H., Cho H.-S., Kim T. (2021). A review of
the porous transport
layer in polymer electrolyte membrane water electrolysis. International Journal of Energy Research.

[20] Padgett E., Bender G., Haug A., Lewinski K., Sun F., Yu H., Cullen D. A., Steinbach A. J., Alia S. M. (2023). Catalyst Layer Resistance
and Utilization in PEM Electrolysis. J. Electrochem.
Soc..

[21] Suermann M., Takanohashi K., Lamibrac A., Schmidt T. J., Büchi F. N. (2017). Influence
of Operating Conditions and Material Properties on the Mass Transport
Losses of Polymer Electrolyte Water Electrolysis. J. Electrochem. Soc..

[22] Weber C. C., Wrubel J. A., Gubler L., Bender G., De Angelis S., Büchi F. N. (2023). How the Porous Transport Layer Interface Affects Catalyst
Utilization and Performance in Polymer Electrolyte Water Electrolysis. ACS Appl. Mater. Interfaces.

[23] Schuler T., Weber C. C., Wrubel J. A., Gubler L., Pivovar B., Büchi F. N., Bender G. (2024). Ultrathin Microporous Transport Layers:
Implications for Low Catalyst Loadings, Thin Membranes, and High Current
Density Operation for Proton Exchange Membrane Electrolysis. Adv. Energy Mater..

[24] Schuler T., Ciccone J. M., Krentscher B., Marone F., Peter C., Schmidt T. J., Büchi F. N. (2020). Hierarchically
Structured Porous
Transport Layers for Polymer Electrolyte Water Electrolysis. Adv. Energy Mater..

[25] Lee J. K., Schuler T., Bender G., Sabharwal M., Peng X., Weber A. Z., Danilovic N. (2023). Interfacial
engineering via laser ablation for high-performing PEM water electrolysis. Applied Energy.

[26] Kang Z., Schuler T., Chen Y., Wang M., Zhang F.-Y., Bender G. (2022). Effects of interfacial
contact under different operating
conditions in proton exchange membrane water electrolysis. Electrochim. Acta.

[27] Liu C., Wrubel J. A., Padgett E., Bender G. (2024). Impacts of PTL coating
gaps on cell performance for PEM water electrolyzer. Applied Energy.

[28] Liu C., Shviro M., Bender G., Gago A. S., Morawietz T., Dzara M. J., Biswas I., Gazdzicki P., Kang Z., Zaccarine S. F. (2023). Degradation Effects
at the Porous Transport Layer/Catalyst Layer Interface in Polymer
Electrolyte Membrane Water Electrolyzer. J.
Electrochem. Soc..

[29] Liu C., Padgett E., Arregui-Mena J. D., Shepherd M., Ware S., Pylypenko S., Cullen D. A., Bender G. (2025). Performance improvement
of proton exchange membrane water electrolysis by surface modification
of porous transport layers. J. Power Sources.

[30] Liu C., Shviro M., Gago A. S., Zaccarine S. F., Bender G., Gazdzicki P., Morawietz T., Biswas I., Rasinski M., Everwand A. (2021). Exploring
the Interface of Skin-Layered Titanium Fibers for Electrochemical
Water Splitting. Adv. Energy Mater..

[31] Liu C., Carmo M., Bender G., Everwand A., Lickert T., Young J. L., Smolinka T., Stolten D., Lehnert W. (2018). Performance
enhancement of PEM electrolyzers through iridium-coated titanium porous
transport layers. Electrochem. Commun..

[32] Hassan N. U., Motyka E., Kweder J., Ganesan P., Brechin B., Zulevi B., Colón-Mercado H. R., Kohl P. A., Mustain W. E. (2023). Effect of porous transport layer
properties on the
anode electrode in anion exchange membrane electrolyzers. J. Power Sources.

[33] Kreider M. E., Maldonado Santos A. R., Clauser A. L., Sweers M. E., Hu L., Volk E. K., Chan A.-L., Sugar J. D., Alia S. M. (2025). Porous
Transport Layers for Anion Exchange Membrane Water Electrolysis: The
Impact of Morphology and Composition. ACS Electrochem..

[34] Park J. E., Choi H. J., Kang S. Y., Jang G. Y., Kim O. H., Karuppannan M., Sung Y. E., Kwon O. J., Cho Y. H. (2022). Effect
of pore structures in nickel-based porous transport layers for high-performance
and durable anion-exchange membrane water electrolysis. International Journal of Energy Research.

[35] Xu Q., Oener S. Z., Lindquist G., Jiang H., Li C., Boettcher S. W. (2021). Integrated
reference electrodes in anion-exchange-membrane
electrolyzers: impact of stainless-steel gas-diffusion layers and
internal mechanical pressure. ACS Energy Letters.

[36] Chen B., Biancolli A. L. G., Radford C. L., Holdcroft S. (2023). Stainless
Steel Felt as a Combined OER Electrocatalyst/Porous Transport Layer
for Investigating Anion-Exchange Membranes in Water Electrolysis. ACS Energy Letters.

[37] Tricker A. W., Ertugrul T. Y., Lee J. K., Shin J. R., Choi W., Kushner D. I., Wang G., Lang J., Zenyuk I. V., Weber A. Z. (2024). Pathways
Toward Efficient and Durable Anion
Exchange Membrane Water Electrolyzers Enabled By Electro-Active Porous
Transport Layers. Adv. Energy Mater..

[38] Razmjooei F., Morawietz T., Taghizadeh E., Hadjixenophontos E., Mues L., Gerle M., Wood B. D., Harms C., Gago A. S., Ansar S. A. (2021). Increasing the performance
of an anion-exchange membrane electrolyzer operating in pure water
with a nickel-based microporous layer. Joule.

[39] Shin S., Park S., Lee Y. R., Shin Y., Yu D. M., Yoon S. J., Jeong H. Y., Kim T.-H., Han H., Sung Y.-E. (2025). Engineering anode porous transport electrode design
for enhanced anion exchange membrane water electrolysis performance. J. Am. Ceram. Soc..

[40] Gangadharan P. K., Kuroki H., Miyanishi S., Okuyama H., Yamaguchi T. (2025). Advanced Surface
Engineering of Porous Transport Electrodes to Improve Membrane–Electrode
Interfaces and Surface Areas for Anion Exchange Membrane Water Electrolyzers. ACS Applied Energy Materials.

[41] Manss-Chmielarz J. J., Morawietz T., Iddon K., Rehse S., Gago A. S., Friedrich K. A. (2025). The Importance
of the Design of Porous Transport Layers:
Unveiling the Interplay Between Structure, Mechanics, and Electrochemistry
in Anion Exchange Membrane Water Electrolysis. Carbon Energy.

[42] Yanagi R., Yang P., Tricker A. W., Chen Y., Scott M. C., Berlinger S. A., Zenyuk I. V., Peng X. (2025). Enhancing water and
oxygen transport through electrode engineering for AEM water electrolyzers. Joule.

[43] Xia L., Gomes B. F., Jiang W., Escalera-López D., Wang Y., Hu Y., Faid A. Y., Wang K., Chen T., Zhao K. (2025). Operando-informed precatalyst
programming towards reliable high-current-density electrolysis. Nat. Mater..

[44] Noh W. Y., Kazmouz S. J., Lee S.-h., Peng J.-K., Shin T. J., Shviro M. (2025). Decoupling electrode kinetics to
elucidate reaction
mechanisms in alkaline water electrolysis. Energy
Environ. Sci..

[45] Yang F., Kim M. J., Brown M., Wiley B. J. (2020). Alkaline Water Electrolysis
at 25 A cm–2 with a Microfibrous Flow-through Electrode. Adv. Energy Mater..

[46] Zhou D., Li P., Xu W., Jawaid S., Mohammed-Ibrahim J., Liu W., Kuang Y., Sun X. (2020). Recent Advances in Non-Precious Metal-Based
Electrodes for Alkaline Water Electrolysis. ChemNanoMat.

[47] Kjartansdóttir C. K., Nielsen L. P., Mo̷ller P. (2013). Development of durable and efficient
electrodes for large-scale alkaline water electrolysis. Int. J. Hydrogen Energy.

[48] Wan L., Xu Z., Xu Q., Pang M., Lin D., Liu J., Wang B. (2023). Key components
and design strategy of the membrane electrode assembly
for alkaline water electrolysis. Energy Environ.
Sci..

[49] Wang J., Zhao Y., Setzler B. P., Rojas-Carbonell S., Ben Yehuda C., Amel A., Page M., Wang L., Hu K., Shi L. (2019). Poly (aryl piperidinium) membranes and ionomers for
hydroxide exchange membrane fuel cells. Nature
Energy.

[50] Alia, S. M. HydroGEN: Low Temperature Electrolysis; Department of Energy: U.S, 2024.

[51] Volk E. K., Kwon S., Alia S. M. (2023). Catalytic Activity
and Stability
of Non-Platinum Group Metal Oxides for the Oxygen Evolution Reaction
in Anion Exchange Membrane Electrolyzers. J.
Electrochem. Soc..

[52] Volk E. K., Clauser A. L., Kreider M. E., Soetrisno D. D., Khandavalli S., Sugar J. D., Kwon S., Alia S. M. (2025). Role of
the Ionomer in Supporting Electrolyte-Fed Anion Exchange Membrane
Water Electrolyzers. ACS Electrochemistry.

[53] Volk E. K., Kreider M. E., Gibson Colón D.
M., Müller M., Sunde S., Alia S. M., Kwon S. (2025). Electrochemical Activation
of Ni–Fe Oxides for the Oxygen Evolution Reaction in Alkaline
Media. ACS Catal..

[54] Pourbaix, M. Atlas of electrochemical equilibria in aqueous solutions; National Association of Corrosion Engineers, 1974.

[55] Volk E. K., Padgett E., Kreider M. E., Kwon S., Alia S. M. (2026). Voltage
breakdown analyses in anion exchange membrane water electrolysis –
the contributions of catalyst layer resistance on overall overpotentials. Energy Adv..

[56] Weiß A., Siebel A., Bernt M., Shen T.-H., Tileli V., Gasteiger H. A. (2019). Impact
of intermittent operation on lifetime and performance
of a PEM water electrolyzer. J. Electrochem.
Soc..

[57] Rajeev M., Jerome-Saboori A., Shekhar R., Boettcher S. W., Kempler P. A. (2025). Impacts of Dissolved Iron on Alkaline Water Electrolysis
Cells. ACS Catal..

[58] Li D., Park E. J., Zhu W., Shi Q., Zhou Y., Tian H., Lin Y., Serov A., Zulevi B., Baca E. D. (2020). Highly quaternized polystyrene ionomers
for high performance
anion exchange membrane water electrolysers. Nature Energy.

[59] Anderson G. C., Pivovar B. S., Alia S. M. (2020). Establishing
Performance Baselines
for the Oxygen Evolution Reaction in Alkaline Electrolytes. J. Electrochem. Soc..

[60] Kreider M. E., Yu H., Osmieri L., Parimuha M. R., Reeves K. S., Marin D. H., Hannagan R. T., Volk E. K., Jaramillo T. F., Young J. L. (2024). Understanding
the Effects of Anode Catalyst
Conductivity and Loading on Catalyst Layer Utilization and Performance
for Anion Exchange Membrane Water Electrolysis. ACS Catal..

[61] Trotochaud L., Young S. L., Ranney J. K., Boettcher S. W. (2014). Nickel–Iron
Oxyhydroxide Oxygen-Evolution Electrocatalysts: The Role of Intentional
and Incidental Iron Incorporation. J. Am. Chem.
Soc..

[62] Stevens M. B., Trang C. D. M., Enman L. J., Deng J., Boettcher S. W. (2017). Reactive
Fe-Sites in Ni/Fe (Oxy)­hydroxide Are Responsible for Exceptional Oxygen
Electrocatalysis Activity. J. Am. Chem. Soc..

[63] Ha M.-A., Alia S. M., Norman A. G., Miller E. M. (2024). Fe-Doped Ni-Based
Catalysts Surpass Ir-Baselines for Oxygen Evolution Due to Optimal
Charge-Transfer Characteristics. ACS Catal..

[64] Becker H., Murawski J., Shinde D. V., Stephens I. E. L., Hinds G., Smith G. (2023). Impact of impurities on water electrolysis: a review. Sustainable Energy & Fuels.

[65] Enman L. J., Burke M. S., Batchellor A. S., Boettcher S. W. (2016). Effects
of Intentionally Incorporated Metal Cations on the Oxygen Evolution
Electrocatalytic Activity of Nickel (Oxy)­hydroxide in Alkaline Media. ACS Catal..

[66] Mauer A. E., Kirk D. W., Thorpe S. J. (2007). The role
of iron in the prevention
of nickel electrode deactivation in alkaline electrolysis. Electrochim. Acta.

[67] Flis-Kabulska I., Flis J. (2014). Hydrogen evolution
and corrosion products on iron cathodes in hot
alkaline solution. Int. J. Hydrogen Energy.

[68] Flis-Kabulska I., Flis J. (2016). Electroactivity of Ni–Fe cathodes in alkaline water electrolysis
and effect of corrosion. Corros. Sci..

[69] Padgett E., Adesso A., Yu H., Wrubel J., Bender G., Pivovar B., Alia S. M. (2024). Performance
Losses and Current-Driven
Recovery from Cation Contaminants in PEM Water Electrolysis. J. Electrochem. Soc..

