# Supplementary material for: Evaluating the Effects of Anode Porous Transport Layer on the Performance and Durability of Anion Exchange Membrane Electrolyzers
Source: ACS Omega. 2026 May 17;11(21):31371–82. doi: 10.1021/acsomega.6c01506 (PMC13234654; doi:10.1021/acsomega.6c01506)
Supplement: Supplementary file 1 [file ao6c01506_si_001.pdf]

Evaluating the effects of anode porous transport layer on the performance and durability of anion exchange membrane electrolyzers

Saad Intikhab[1,#], Alexandra Oliveira[2,$], Kimberely S. Reeves[3], Haoran Yu[3], Yushan Yan[2], Shaun M. Alia[1,*]

[1] Chemical and Material Sciences Center, National Laboratory of the Rockies, 15013 Denver West Parkway, Golden, Colorado, 80401, United States of America

[2] Department of Chemical and Biomolecular Engineering, University of Delaware, 221 Academy Street, Newark, Delaware, 19716, United States of America

[3] Center for Nanophase Materials Sciences, Oak Ridge National Laboratory, 1 Bethel Valley Road, Oak Ridge, Tennessee, 37830, United States of America

Current addresses:
[#] Honeywell, 201 W Oakton Street, Suite C, Des Plaines, Illinois, 60018, United States of America
[$] Mott Corporation, 84 Spring Lane, Farmington, Connecticut, 06032, United States of America

* Corresponding author email: shaun.alia@nlr.gov

Supporting Information

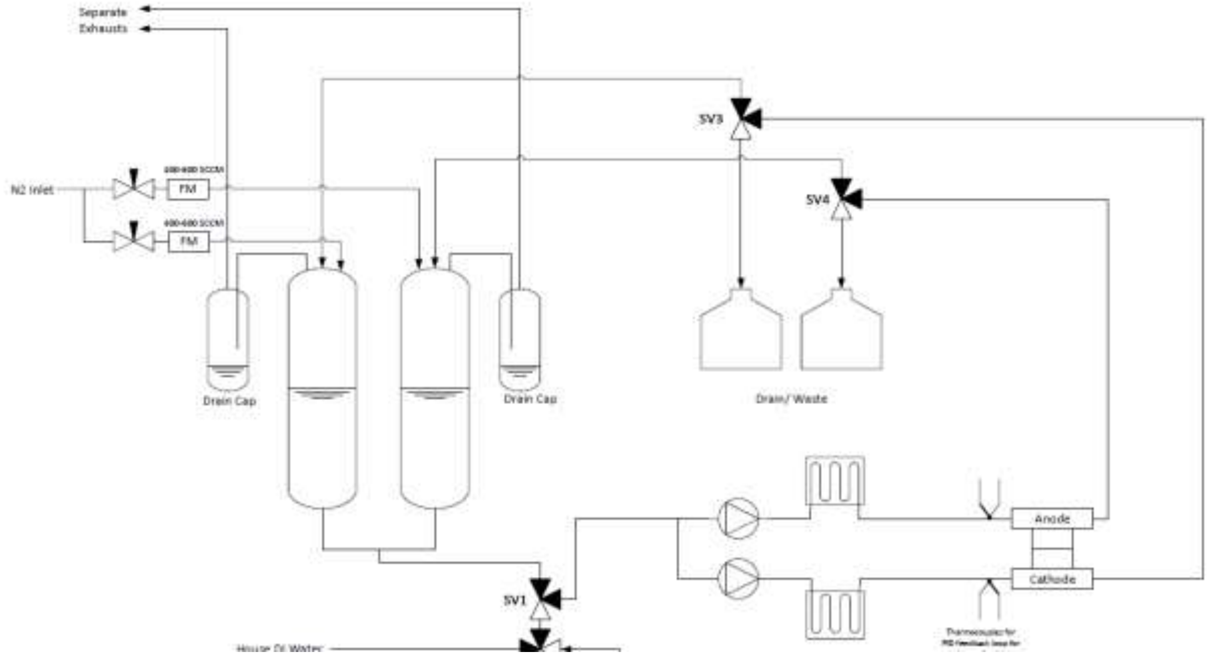

Figure S1: P&ID of AEM test station.

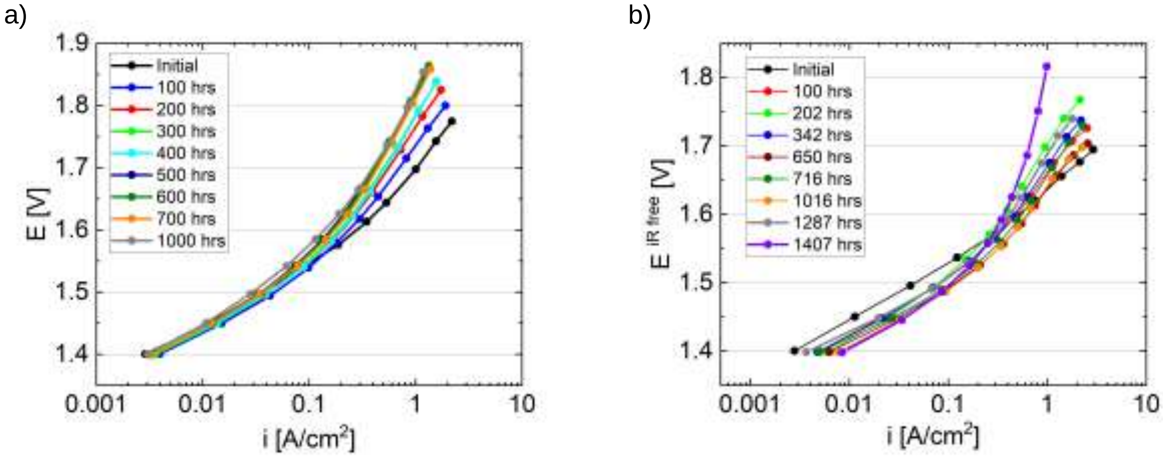

Figure S2: Tafel plot as a function of time for MEAs with anode PTL: (a) Ni PTL; (b) SS PTL.

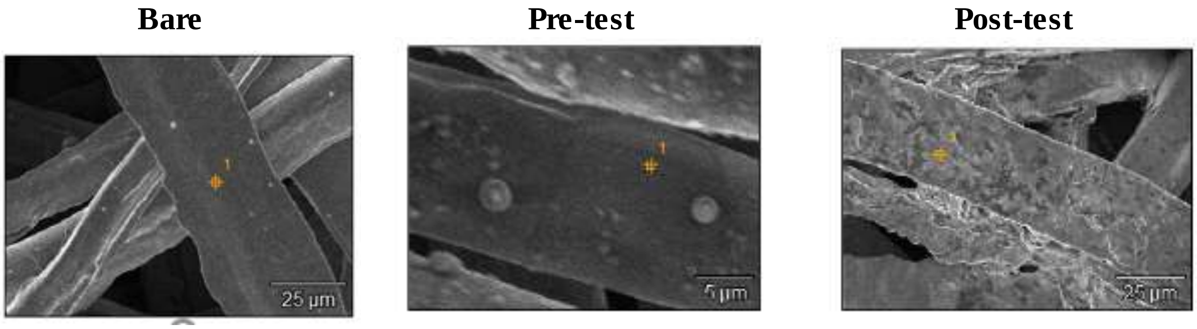

Figure S3: SEM images of bare Ni PTL; Ni PTL sprayed with $NiFeO_x$ catalyst pre-test and post-durability test for EDS.

Table S1: EDS elemental analysis of Ni PTL (bare, pre-test and post-test).

**Ni PTL**	**C**	**O**	**Fe**	**Ni**	**K**
**Bare (wt%)**	1.385	0.662	0	97.954	0
**Pre-test (wt%)**	31.346	17.528	38.032	13.094	0
**Post-test (wt%)**	2.125	29.192	28.763	30.406	9.515

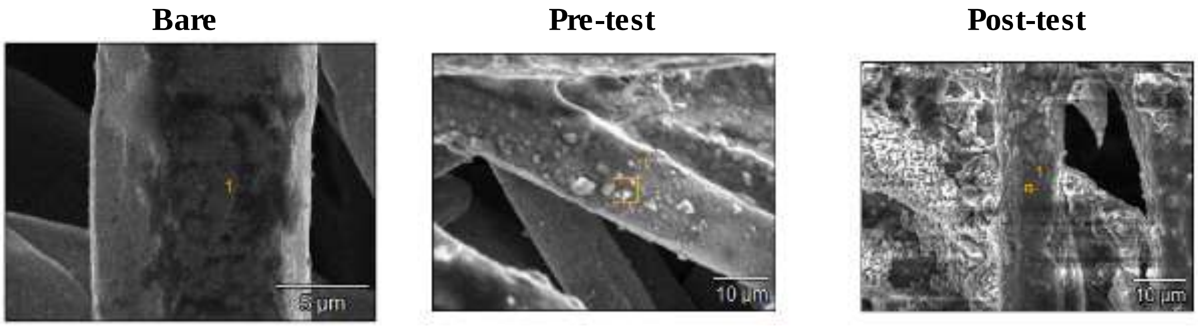

Figure S4: SEM images of bare SS PTL; SS PTL sprayed with $NiFeO_x$ catalyst pre-test and post-durability test for EDS.

Table S2: EDS elemental analysis of SS PTL (bare, pre-test and post-test).

**SS PTL**	**C**	**O**	**Fe**	**Ni**	**K**
**Bare (wt%)**	3.358	5.337	72.634	18.277	0
**Pre-test (wt%)**	31.067	19.435	33.688	15.810	0
**Post-test (wt%)**	3.612	30.427	20.008	33.203	12.751

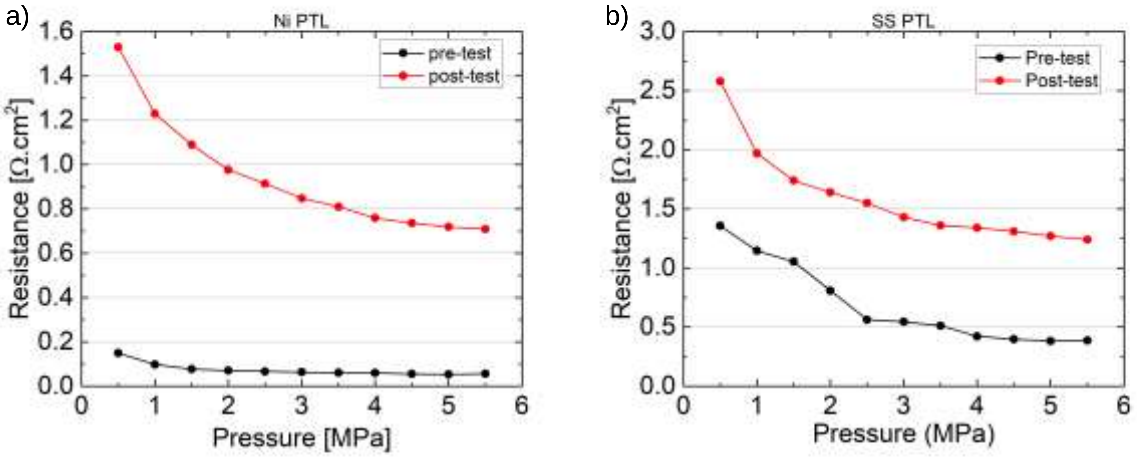

Figure S5: Interfacial contact resistance (ICR) measurements for sprayed (a) Ni PTL and (b) SS PTL pre-test and post- durability test. The electronic resistance (ex situ) increases significantly for both PTLs indicating oxidation of the PTL. The HFR measurement (end-of-test) in situ reflects some of this change however, the change is not as drastic as seen ex situ. The ex situ measurement is conducted at room temperature, compressing the anode PTL between gold plates at different pressures and measuring the voltage at 1 A.

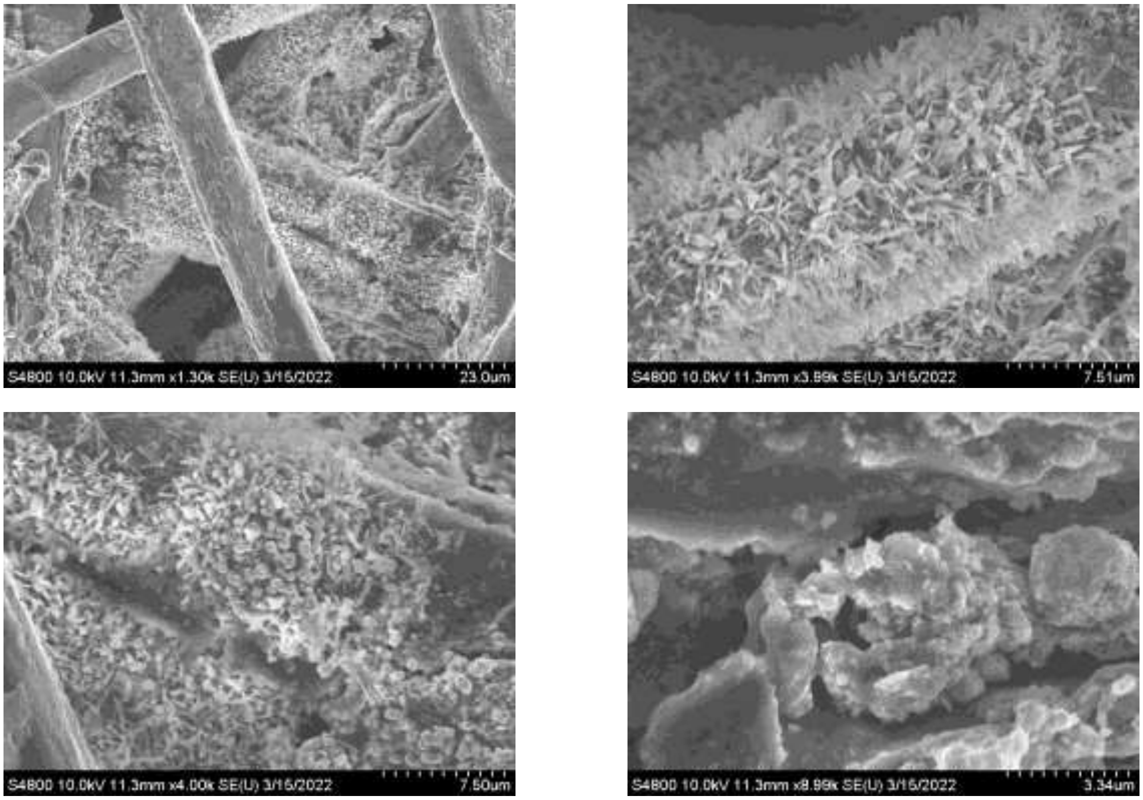

Figure S6: SEM images of SS PTL post-test.

Table S3. EDS elemental analysis of pre-test NiFeOx from SS PTL, post-test NiFeOx from Ni PTL, and post-test NiFeOx from SS PTL.

	**O [at %]**	**Fe [at %]**	**Ni [at %]**	**Fe/Ni [Atomic Ratio]**
Pre-test	74.4±1.0	16.2±0.6	9.4±0.7	1.73
Post-test, Ni PTL	76.8±0.7	11.4±0.7	11.8±0.4	0.97
Post-test, SS PTL	74.3±2.5	17.3±1.3	8.4±1.6	2.04
